# Activation of alkyl hydroperoxides by manganese complexes of tmtacn for initiation of radical polymerisation of alkenes†

**DOI:** 10.1039/d4cy00042k

**Published:** 2024-03-13

**Authors:** Linda E. Eijsink, Andy S. Sardjan, Esther G. Sinnema, Hugo den Besten, Yanrong Zhang, Ronald Hage, Keimpe J. van den Berg, Jitte Flapper, Ben L. Feringa, Wesley R. Browne

**Affiliations:** a Stratingh Institute for Chemistry, University of Groningen Nijenborgh 4 9747 AG Groningen the Netherlands w.r.browne@rug.nl; b Akzo Nobel Car Refinishes N.V Rijksstraatweg 31 2171 AJ Sassenheim the Netherlands; c Akzo Nobel Decorative Coatings B.V Rijksstraatweg 31 2171 AJ Sassenheim the Netherlands

## Abstract

The activation of alkyl hydroperoxides to generate radicals is a key step in the initiation of radical polymerisations in many industrial applications, not least protective coatings. Cobalt soaps (Co(ii) alkyl carboxylates) are highly effective catalysts under ambient conditions but viable alternatives based on less scarce catalysts are desirable, with especially iron and manganese catalysts showing potential. Manganese complexes of the ligand *N*,*N*′,*N*″-trimethyl-1,4,7-triazacyclononane (tmtacn) are long established as catalysts for organic oxidations with H_2_O_2_, however their reactivity with alkyl hydroperoxides is less studied especially in apolar solvents. Here we show that this family of complexes can be employed as catalysts for the decomposition of alkyl hydroperoxides in apolar solvents such as styrene/methyl methacrylate mixtures and resins based on styrene/bisphenol-A based diglycidyl ether bismethacrylate (BADGE-MA). The progress of alkene polymerisation in crosslinking resins is followed by Raman spectroscopy to establish its dependence on the oxidation state of the manganese catalyst used, as gelation time and onset of autoacceleration are of particular interest for many applications. We show, through reaction progress monitoring with UV/vis absorption and Raman spectroscopy, that the stability of the manganese complexes in the resin mixtures has a substantial effect on curing progress and that the oxidation state of the resting state of the catalyst is most likely Mn(ii), in contrast to reactions with H_2_O_2_ as oxidant in which the oxidation state of the resting state of catalyst is Mn(iii). Manganese complexes of tmtacn are shown to be capable initiators of alkene radical polymerisations, and their rich coordination and redox chemistry means that resin curing kinetics can potentially be tuned more readily than with cobalt alkyl carboxylates.

## Abstract 2

Catalysed activation of alkyl hydroperoxides to generate radicals is a key step in the initiation of radical polymerisations in many applications, not least protective coatings. Manganese complexes show potential as replacements for cobalt catalysts.

## Graphical abstract

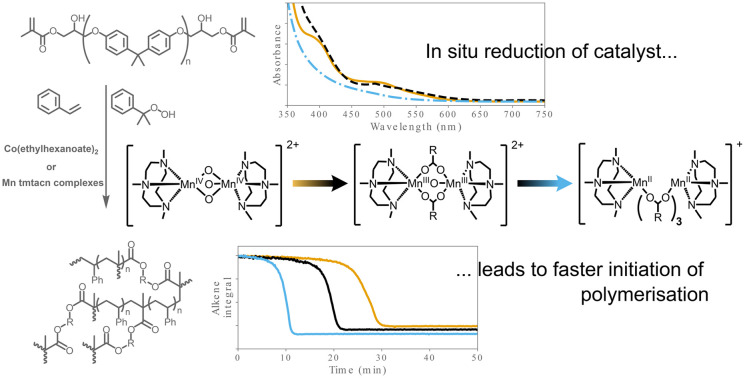

## Introduction

Reactive oxygen species are a remarkable class of compounds that are central to the modern chemical industry, from pharmaceuticals and fine chemical production^1^ to initiation of polymerisation in resins and coatings.^2,3^ For practical purposes, stable oxidants are necessary to allow for purification, transport and storage. Molecular oxygen is the oxidant of choice due to its ubiquity, however, the activation of O_2_ is not always feasible and in these cases peroxides take the lead, with H_2_O_2_ being the most atom economic.^1,4–7^ For specific applications, peracids, alkyl and benzyl (hydro)peroxides are preferred. These oxidants are stable and require activation at point of use, with thermal activation, *e.g.*, in radical based curing of alkene based resins, the simplest but least controllable approach. Catalysts, especially transition metal ion alkyl carboxylates, can activate organic (hydro)peroxides for application under ambient conditions.^2,8,9^ The reactivity and outcome achieved with catalysts are highly condition dependent and extrapolation of behaviour and selectivity from a simple solvent to a complex resin mixture is challenging. In the case of curing of alkene based resins, cobalt alkyl carboxylates, the main catalysts in current use, were likely chosen due to the success achieved with cobalt in replacing lead salts as catalyst in alkyd paints, in which they activate molecular oxygen and the alkyl peroxides formed *in situ*.^2,10^ The current drive to replace cobalt due to concerns over safety presents the challenge to find catalysts that can activate alkyl hydroperoxides in the currently used alkene resin formulations with the same curing profile (delay before onset of autoacceleration^11^ to allow for application) as obtained with cobalt.^2,3,12–14^

Manganese alkyl carboxylates can catalyse the breakdown of alkyl peroxides to generate radicals needed to initiate curing and are considered a potential replacement for cobalt alkyl carboxylates.^2,3^ However, the lag-period before the start of the autoacceleration phase in styrene/bismethacrylate based resins is too long to be useful in most applications.^14^ Approaches to control catalyst performance include adding ligands together with manganese alkyl carboxylates, or using well-defined complexes. Such approaches have been taken in the curing of alkyd based coatings (in which O_2_ is activated to form alkyl peroxides *in situ*).^2,13^ Manganese complexes of alkyl amine and alkyl pyridyl ligands have proven to be a diverse and robust family of catalysts for the activation of peracids and H_2_O_2_, especially towards alkene, alcohol, aldehyde and alkane C–H oxidation.^5–7,16^ For fine chemical production, most academic efforts have focused on oxidations in CH_3_CN and water, with a particular focus on activation of peracids and H_2_O_2_ to generate reactive high valent complexes that engage in hydrogen atom abstraction and oxygen atom transfer reactions. The activation of alkyl (hydro)peroxides, especially by manganese complexes,^17^ has received much less attention in part due to their reduced atom economy and tendency to form alkoxy radicals. Nevertheless, alkylperoxides have been applied to organic oxidations with some success.^18–20^ Amongst the various classes of manganese complexes, those of the triazacyclononane family, and especially *N*,*N*′,*N*″-trimethyl-1,4,7,-triazacyclononane (tmtacn), have received considerable attention for activation of H_2_O_2_ over the last three decades.^16,21,22^

**Fig. 1:**
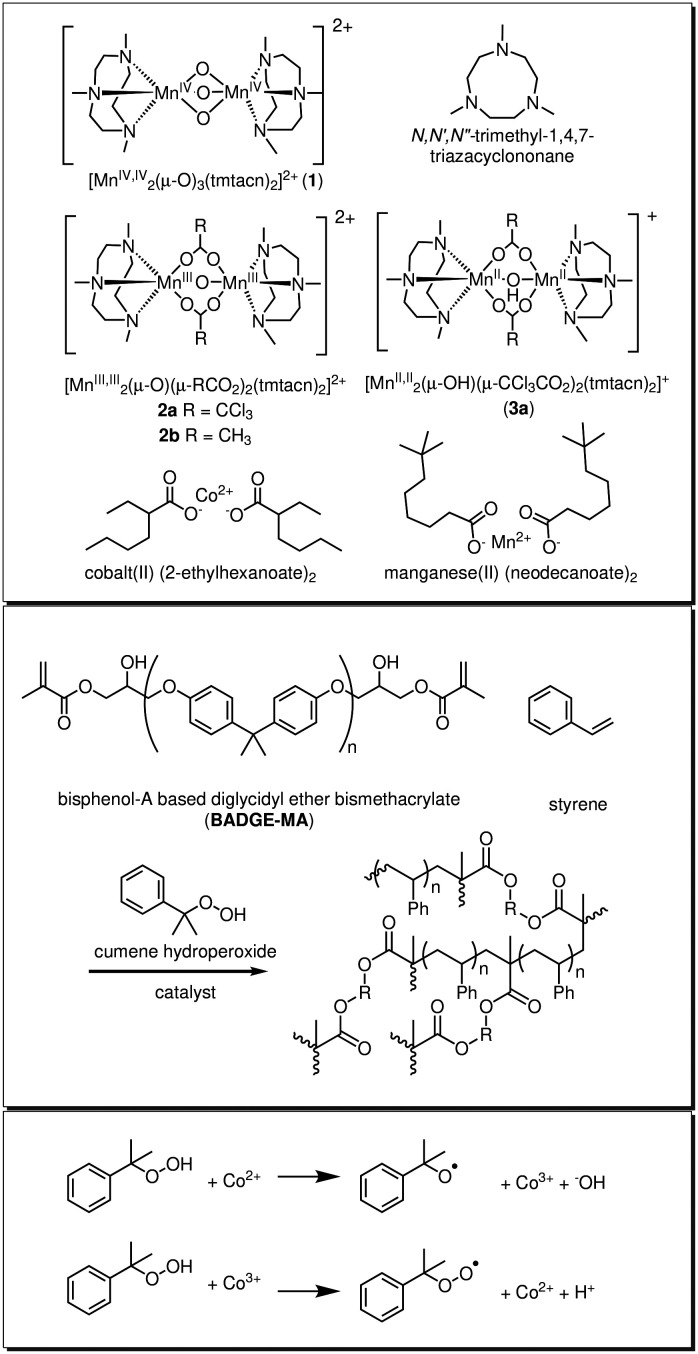
Chemical structures of (upper panel) the manganese complexes 1, 2a, 2b, and 3a and the ligand *N*,*N*′,*N*″-trimethyl-1,4,7-triazacyclononane (tmtacn).^15^ Described in the text, of cobalt and manganese alkyl carboxylates, (middle panel) styrene and BADGE-MA polymerisation initiated by cumene hydroperoxide and a catalyst, and (bottom panel) formation of alkoxy and peroxy radicals from cumene hydroperoxide.

Manganese complexes of tmtacn show a diverse oxidation state dependent coordination chemistry, with isolated mono- and dinuclear complexes ranging from the Mn(ii) to Mn(iv) oxidation states (Fig. 1).^23–27^ The Mn(iv) dinuclear complex [Mn^IV,IV^_2_(μ‐O)_3_(tmtacn)_2_]^2+^ (1) was first reported by Wieghardt and coworkers in the 1980s^26^ and is applied commercially in dishwasher powder formulations.^21^ More recently their industrial application has expanded to include curing of alkyd paints (activation of molecular oxygen) and decomposition of alkyl hydroperoxides formed during curing.^3,10,13^ This latter reactivity is of particular interest as it indicates that such complexes can activate alkyl hydroperoxides and hence have application in curing of alkene based resins, *e.g.*, styrene/(bis-)methacrylate mixtures.^14^

Here, we show that the alkyl hydroperoxides can be activated by manganese complexes of the ligand tmtacn to trigger radical initiated alkene polymerisation. The structure and oxidation state of the complex(es) responsible for generating radicals from cumene hydroperoxide in alkene based resin is investigated. It is shown that well defined complexes, such as 1, can provide for predictable curing behaviour, which, in contrast to cobalt and manganese alkyl carboxylates, can be tuned by the form of the complex used. However, we show also that the various complexes used are precursors to the active form of the catalyst, which itself can be generated *in situ* by mixing Mn(neodecanoate)_2_ with the tmtacn ligand.

## Experimental

### Materials

Styrene, toluene, methyl methacrylate, cumene hydroperoxide (80% solution in cumene), Al_2_O_3_ 90 active 70–230 mesh, Co(ii)(2-ethylhexanoate)_2_ (65 wt% of complex in mineral spirits), and Mn(ii)(neodecanoate)_2_ (8 wt% of manganese in mineral spirits) were obtained from commercial sources and used as received. *N*,*N*′,*N*″-Trimethyl-1,4,7-triazacyclononane (tmtacn), [Mn^IV,IV^_2_(μ‐O)_3_(tmtacn)_2_](PF_6_)_2_ (1) and [Mn^III,III^_2_(μ‐O)(CH_3_CO_2_)_2_(tmtacn)_2_](PF_6_)_2_ (2b) were obtained from WeylChem. The synthesis and characterisation of bisphenol-A based diglycidyl ether bismethacrylate (BADGEMA) was reported elsewhere.^14^ The ligand and complexes (2a, 3a)^25,26^ used in this study were available from earlier studies.^15^ Mixtures of Mn(neodecanoate)_2_ and the tmtacn were prepared by direct addition to mixtures of styrene/BADGE-MA (Fig. 1). Manganese complexes of the ligand tmtacn including 1, 2a, 2b, and 3a (Fig. 1), were added to the reaction mixtures as acetonitrile solutions.

### Spectroscopy

UV/vis absorption spectra were recorded using a Specord S600 or S210plus spectrophotometer (Analytik Jena) in 2 mL glass vials. Raman spectra were recorded using either: (i) a home-built Raman probe at 785 nm; briefly an Ondax 785 Surelock RO module (75 mW) was reflected by a long pass dichroic (Semrock Brightline Di02-R785-25) to a 30 cm focal length planoconvex lens and the Raman scattering collected at 180° (backscattering geometry) through the dichroic and a long pass filter (Semrock Brightline BLP01-785R-25, with 50% cut-off at 805 nm, 309 cm^−1^) and fed to a 100 micron round to line bundle fiber by a Thorlabs SMA fiber port (PAF2S-A7B). Spectra were collected using an Andor Technology Shamrock163i spectrograph equipped with a 600 l mm^−1^ 830 nm blazed grating iDus 420-OE CCD using an external trigger. Multiple samples were measured in sequence using a custom built Quantum Northwest 6-position cell changer equipped with optical access and temperature control. Samples were held at 25 °C over the course of the reaction. The software T-app (Quantum Northwest) was used to control sample position and trigger the spectrometer. T-app also provided timestamps over the course of the measurement, or (ii) using a Cobolt laser (785 nm, 500 mW), a fibre-coupled InPhotonics probe head and a Shamrock163i spectrograph equipped with a 600 l mm^−1^ 830 nm blazed grating and an iVac-324B-FI-560 CCD (Andor Technology) and QNW FLASH temperature controlled cuvette holder. Spectra were calibrated with cyclohexane (ASTM E1840 96-R22) and spectra processed using either Spectragryph 16.1 or Python. A DS18B20 waterproof temperature immersion sensor was used where temperature was recorded directly in samples.

### Curing of styrene/BADGE-MA resin monitored by Raman spectroscopy

The conversion of alkene during polymerisation of resin mixtures was followed over time by Raman spectroscopy through the change in intensity of the C=C stretching bands of styrene and BADGE-MA at 1630 and 1637 cm^−1^, respectively.^14^ The integrated area of these Raman bands corresponds inversely with the extent of alkene polymerisation. Spectra were normalised using the band at 1118 cm^−1^ following a single point baseline correction at 1070 cm^−1^. In a typical experiment, batches of resin were prepared by mixing 10 g of BADGE-MA containing 10 mg (45.4 μmol) of butylated hydroxytoluene with 3.4 g (3.75 mL) of styrene unless stated otherwise. The stabiliser present in the styrene was removed by filtration over neutral aluminium oxide prior to use. Catalysts were added as concentrated solutions in styrene (Co(ii)(2-ethylhexanoate)_2_ and Mn(neodecanoate)_2_), or in acetonitrile (1, 2b, 2a and 3a), to 2 mL of the resin and mixed thoroughly using an orbital vortex mixer. 228 μL (235 mg, 1.23 mmol) of cumene hydroperoxide (80% in cumene) was added to initiate the reaction and samples were again mixed briefly using an orbital vortex mixer before placing in the temperature controlled vial holder.

### Cumene hydroperoxide determination by iodine liberation

Cumene hydroperoxide concentrations were determined through oxidation of iodide to iodine, using literature procedures.^28,29^ 500 μl aliquots were withdrawn from 18 ml of curing BADGE-MA/styrene resin, or methyl methacrylate/styrene mixtures, and added to a flask containing 4 ml of saturated NaI in isopropyl alcohol (IPA) and 10 ml acidified IPA (10% acetic acid v/v). After heating at reflux for a minimum of 5 min the brown solution was cooled to room temperature before adding 2 ml of demineralized water. The solution was diluted 20 fold and its UV/vis absorption spectrum was recorded on a Specord210plus (Analytik Jena) spectrophotometer using a 2 mm pathlength quartz cuvette. The concentration of triiodide (I_3_^−^) was determined using a calibration curve of known cumene hydroperoxide concentrations in the model mixture. For each measurement the blank was determined by measuring a sample prior to addition of cumene hydroperoxide.

## Results

Earlier studies by our group and others focused on oxidation of organic substrates with H_2_O_2_ catalysed by 1, 2a/3a and 2b in solvents such as acetonitrile and acetone.^5,15,23^ Oxidations with these and analogous complexes using alkyl peroxides have been noted occasionally (*vide supra*),^20,30–32^ however, these polar solvents are not representative of the relatively apolar styrene/BADGE-MA mixtures of interest in the present study, and hence the complexes' behaviour cannot readily be extrapolated to the later matrix. Indeed, the most direct evidence for the activation of cumene hydroperoxide by complexes 1 and 2b under conditions relevant for alkene polymerisation can be obtained from the products formed in apolar solvents, and especially those based on alkenes. Therefore their reactivity in styrene, toluene, and mixtures of styrene/methyl methacrylate (as a model for alkene resins to be studied) was explored first with 1 or 2b, as they are air and moisture stable.

**Fig. 2:**
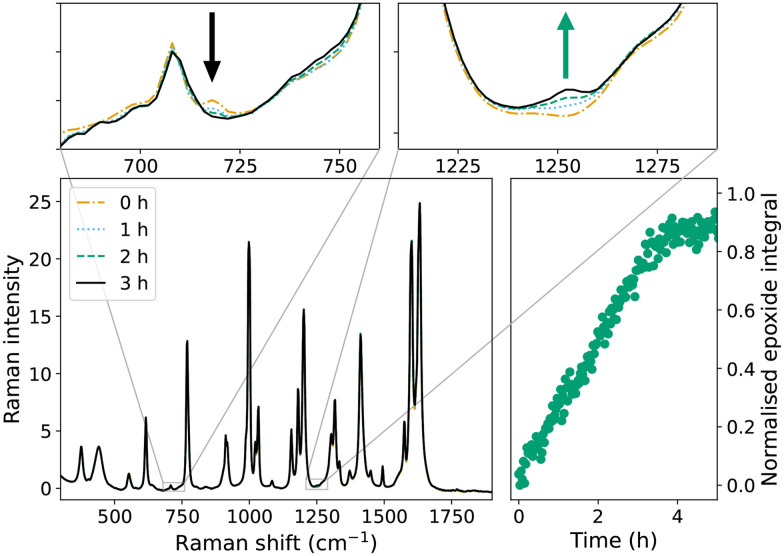
Decomposition of cumene hydroperoxide catalysed by 2b in styrene over 3 h monitored by Raman spectroscopy. The insets show characteristic bands of the hydroperoxide (*ν*_O–Ostr_ at 718 cm^−1^, black arrow) consumed and of the styrene oxide (*ν*_C–H in-plane-bend_ at 1252 cm^−1^, green arrow) formed.

Addition of cumene hydroperoxide to neat styrene, with either 1 or 2b, yields styrene oxide (1252 cm^−1^, Fig. 2), consistent with epoxidation of alkenes by these catalysts with H_2_O_2_ reported earlier,^15,21,33^ and indicates that these complexes are catalytically active with cumene hydroperoxide as terminal oxidant. However, although styrene oxide formation indicates catalyst activation of cumene hydroperoxide, it does not provide direct evidence for the generation of the radical species needed to initiate radical chain polymerisation. In this regard, toluene provides an apolar solvent environment and, in contrast to styrene, gives an indication of the activation of alkyl peroxides to generate radical species since the oxidation of toluene is typically *via* an initial hydrogen atom abstraction step.^18,34^ Addition of cumene hydroperoxide to a solution of 2b in toluene shows formation of benzaldehyde (band at 1705 cm^−1^, Fig. S1 and S2†), indicating significant C–H oxidation activity, and hence generation of intermediate radical species.

**Fig. 3:**
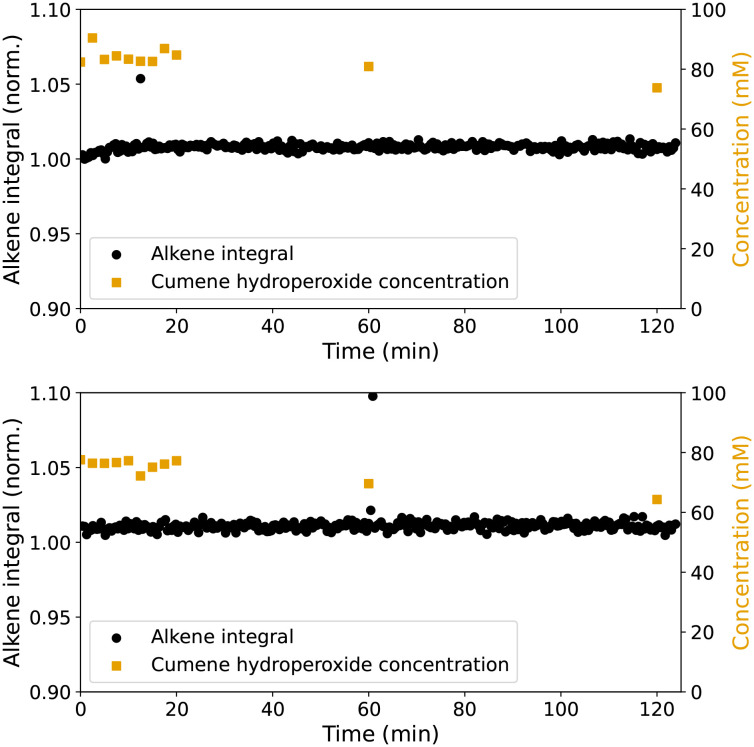
Cumene hydroperoxide concentration (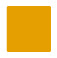, initial concentration 87 mM) over time, determined by iodine liberation,^28,29^ in styrene/methyl methacrylate with Mn(neodecanoate)_2_ (top) and 2b (bottom), shown with the area (alkene integral normalised to initial intensity) of the alkene C=C stretch band at 1630–1637 cm^−1^ (●, averaged over 5 spectra).

A mixture of styrene and methyl methacrylate (MMA) was used as a model for the alkenes present in alkene resins discussed below. Given the known reactivity of 1 and 2b it is expected that styrene would undergo epoxidation as observed with styrene alone (*vide supra*, the catalysts show greater reactivity with electron rich alkenes with H_2_O_2_ as terminal oxidant).^15^ The concentration of cumene hydroperoxide, determined by titration, in the styrene/MMA mixture decreases by <20% over the first hour with 2b at a rate marginally higher than with Mn(neodecanoate)_2_, Fig. 3, showing that both catalysts activate the initiator. However, styrene oxide is not observed as a product (Fig. S3†) and instead the minor decrease in styrene concentration, consistent with some polymerisation, is observed. The low alkene conversion for the solution phase (co-)polymerisation of styrene and methyl methacrylate is expected due to inhibition by chain termination reactions.^35^

Introduction of a cross-linking methacrylate component greatly enhances the rate and extent of polymerisation (through autoacceleration) and thus the impact of cumene hydroperoxide decomposition by the catalysts should be more pronounced.

### Radical polymerisation of styrene/BADGE-MA

**Fig. 4:**
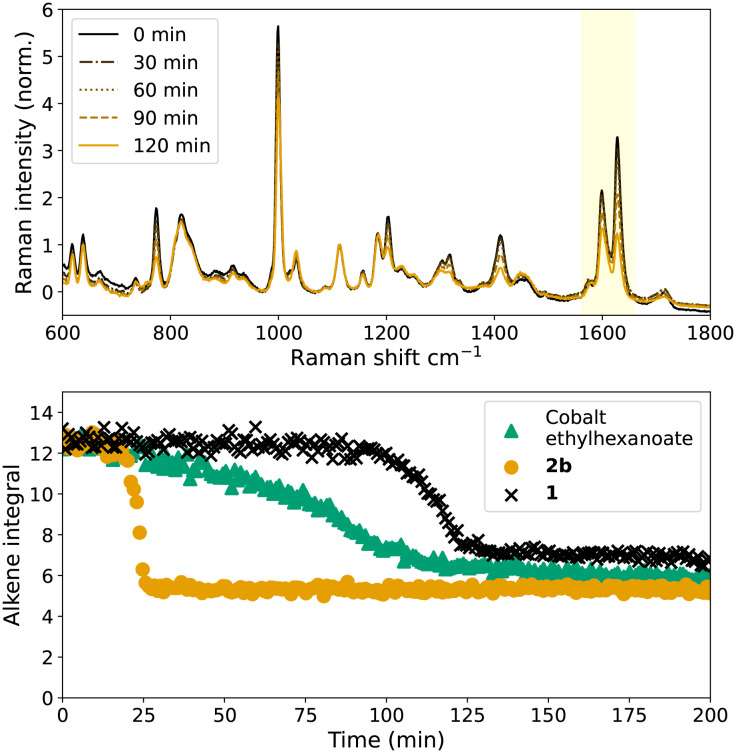
Polymerisation of styrene/BADGE-MA initiated by catalysed decomposition of cumene hydroperoxide; (top) selected spectra for reaction with Co(ii)(2-ethylhexanoate)_2_ (bottom) time dependence of alkene polymerisation by Co(ii)(2-ethylhexanoate)_2_ (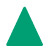), 2b (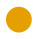), and 1 (x) followed by Raman spectroscopy (*λ*_exc_ 785 nm) monitored by area (alkene integral) of the alkene C=C stretch band at 1630–1637 cm^−1^.

**Fig. 5:**
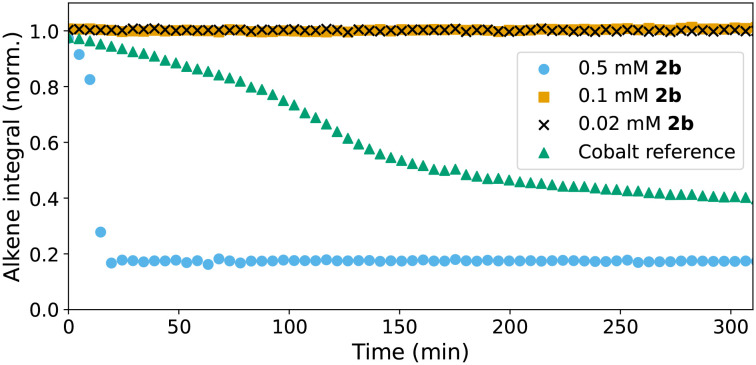
Cumene hydroperoxide initiated polymerisation with 0.5 mM (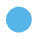), 0.1 mM (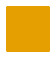), and 0.02 mM (x) 2b. Polymerisation with Co(ii)(2-ethyl hexanoate)_2_/cumene hydroperoxide is shown for reference (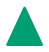).

The time course of polymerisation of mixtures of styrene/BADGE-MA (Fig. 4) initiated by catalytic or thermal decomposition of cumene hydroperoxide can be determined *in situ* by Raman spectroscopy, manifested in the change in intensity of the alkene stretching bands, as well as other changes in the spectrum characteristic of the loss of conjugation that accompanies polymerisation.^14^ The time dependence of alkene conversion/polymerisation during curing of BADGE-MA/styrene mixtures initiated with cumene hydroperoxide with either 1, or 2b as catalyst, Fig. 4 shows that both catalysts provide for sigmoidal shaped reaction profiles^36^ as observed for the standard catalyst Co(ii)(2-ethylhexanoate)_2_. All three catalysts show a period of little or no conversion of alkene followed by an autoacceleration phase until a glass is formed at which point the polymerisation of remaining alkene effectively stops. The lag period before the onset of autoacceleration is due in part to the inhibition of polymerisation due to, *e.g.*, oxygen and BHT (Fig. S4†), however, even when consumed the polymerisation is limited by termination events until the resin reaches a state where the growing chain ends can no longer meet. Thereafter the polymerisation accelerates until the viscosity is such as to limit diffusion and the polymerisation effectively halts. It should be noted that styrene polymerisation continues, albeit much more slowly thereafter. While some conversion is observed with Co(ii)(2-ethylhexanoate)_2_ during the initial ‘slow’ phase, for the manganese catalysts conversion is not observed until shortly before the onset of the autoacceleration phase. Furthermore, all catalysts show much shorter lag periods than with Mn(neodecanoate)_2_, *vide infra*, *in situ* catalyst preparation. The duration of the lag phase for the two manganese complexes is substantially different: the lag period with 1 is typically >90 min, whereas with 2b autoacceleration is observed within *ca.* 30 min. Furthermore, although reaction progress shows little sensitivity to the concentration of Co(ii)(2-ethylhexanoate)_2_ (Fig. S5†),^14^ the reaction rate is highly sensitive to the concentration of 2b, Fig. 5, with no reaction at 0.02 and 0.1 mM but rapid reaction rate at 0.5 mM.

The lack of reactivity before the sudden onset and autoacceleration of polymerisation suggests fundamental differences in their reactivity compared to Co(ii)(2-ethylhexanoate)_2_ and Mn(neodecanoate)_2_. The difference in time before the onset of alkene conversion with 1 compared to 2b and the lack of conversion until shortly before auto-acceleration begins, indicates that both of the catalysts need to undergo activation, *i.e.* a change in structure. A lag period with no activity followed by a sudden change in oxidation state (*vide infra*) and structure coinciding with the onset of catalytic activity was observed for 1 earlier in the epoxidation of alkenes with H_2_O_2_.^15,23^ Under those conditions, the lag period was highly reproducible, but its length was dependent on the presence and type of carboxylic acid (RCO_2_H) present. The origin of the lag period was shown to be due to an autocatalytic transformation of 1 to [Mn^III,III^_2_(μ‐O)(RCO_2_)_2_(tmtacn)_2_]^2+^ complexes.^23,37^ It is of note that the duration of the lag time was also sensitive to (shortened by) the presence of impurities, such as reductants or manganese complexes in oxidation states lower than the +IV oxidation state (*vide infra*). It should be noted that although the reductant butylated hydroxytoluene (BHT) is present in the styrene/BADGE-MA resin used,^14,38^ BHT alone will not reduce 1, as it is not a sufficiently strong reductant.^39^ However, the BADGE-MA resin contains *ca.* 0.1 mmol acid per gram, providing conditions for formation of species such as [Mn^III,III^_2_(μ‐O)(RCO_2_)_2_(tmtacn)_2_]^2+^, which occurs slowly on standing (*vide infra*).^15,21,23,40^ It should be noted that removal of BHT and residual acid from BADGE-MA is synthetically impractical.

**Fig. 6:**
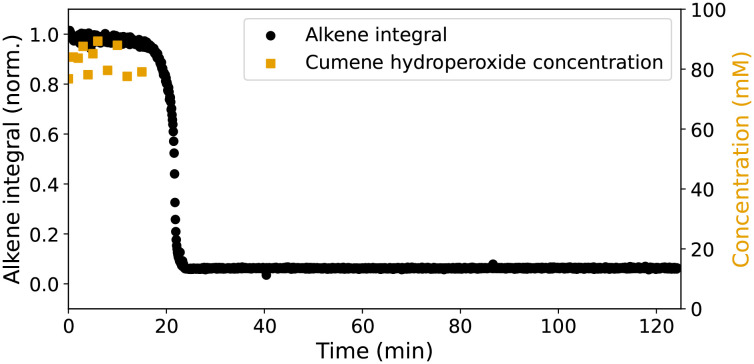
Cumene hydroperoxide concentration (initial: 87 mM, 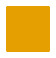) over time, monitored by iodine liberation,^28,29,41^ in styrene/BADGE-MA resin with 2b. The intensity of Raman (*λ*_exc_ 785 nm) band of alkene C=C stretching mode (at *ca.* 1615 cm^−1^, ●) is shown.

In styrene/MMA (*vide supra*), 2b gave little if any decomposition of cumene hydroperoxide over the first 2 h. Similarly, little decomposition was observed in styrene/BADGE-MA over the first 15 min (*i.e.* until just before the gel point), Fig. 6. It should be noted that it is not possible to follow the concentration of cumene hydroperoxide beyond the gel-point of these resins by titration (the gel-point is reached when the concentration of polymer is sufficient to form a mechanically stable organogel).

Although it is clear that both 1 and 2b activate the initiator cumene hydroperoxide, with lag periods that are comparable with that provided by Co(ii)(2-ethylhexanoate)_2_, several differences between the performance of the catalysts are immediately noticeable (Fig. 5). Whereas polymerisation of alkene is apparent with Co(ii)(2-ethylhexanoate)_2_, conversion is not observed with 1 and 2b until shortly before the onset of the autoacceleration phase. However, the lag period for 1 is longer and for 2b shorter than for Co(ii)(2-ethylhexanoate)_2_, and although the extent of alkene polymerisation is similar for all three catalysts, higher conversions can be observed depending on conditions (*vide infra*). Furthermore, despite the lack of alkene polymerisation before the onset of the autoacceleration phase for both 1 and 2b, with both catalysts the maximum rate of polymerisation is much higher than with Co(ii)(2-ethylhexanoate)_2_. It should be noted that the vibrational spectra of the cured resins formed with all of the catalysts are essentially the same and show characteristic changes of the loss of alkene bonds and formation of alkanes.^14^ Moreover, in all cases hard insoluble solids are obtained as expected for a heavily crosslinked polymer. The origins of these differences in behaviour are explored further, in particular the impact of reaction exotherm on conversion and reaction rate, before the origin of differences in lag period observed for 1 and 2b and the nature of the active form of the catalysts is assessed.

### Heat of polymerisation, reaction rate, and extent of conversion

The *T*_g_ of the styrene/BADGE-MA resins when cured depends on the extent of alkene conversion, which in turn depends on temperature. The maximum extent of alkene conversion in styrene/BADGE-MA mixtures is reached when the *T*_g_ of the curing resin reaches the temperature of the sample, *i.e.* a glassy state is reached and further propagation is limited by the inability of the monomer to diffuse to the alkyl radical bearing ends of the polymer chains.^42^ With Co(ii)(2-ethylhexanoate)_2_ as catalyst, the extent of alkene polymerisation correlates linearly with the maximum temperature at which the sample is held, either during initial curing or post curing, Fig. S6.†^14^ Although the temperature of samples (*i.e.* 2 mL vials) was controlled using a thermostated sample holder, the low thermal conductivity of the resins means that the loss of heat to the surroundings is insufficient to adequately remove the heat generated by the exothermic reaction (alkene polymerisation).

Direct measurement of the temperature within the bulk of the resin using a thermistor with simultaneous monitoring of conversion by Raman spectroscopy (focused near the thermistor) during curing with Co(ii)(2-ethylhexanoate)_2_ or 2b shows that with Co(ii)(2-ethylhexanoate)_2_, the reaction temperature stays within 2 °C of the set temperature of the sample holder, while with 2b, the temperature of the resin increases to almost 10 °C above the set temperature (Fig. S7†). It is notable that the sample temperature continues to increase until the glass point (where conversion halts) is reached and thereafter cools. The temperature increase is due to the reaction exotherm coupled with the poor thermal conductivity of the resin, and once polymerisation halts due to the rigidification (glass like state), heat is no longer released and the sample cools. The conversion in the latter case corresponds to the conversion expected with Co(ii)(2-ethylhexanoate)_2_ at 36 °C (*vide supra*) and the temperature reached by the resin depends both on the rate of polymerisation (exothermic reaction) during the autoacceleration phase and the loss of heat from the sample to the environment. Therefore the extent of conversion can be taken as an indication of the maximum temperature reached by the sample.

### UV/vis absorption spectroscopy of manganese complexes in styrene/BADGE-MA resin

**Fig. 7:**
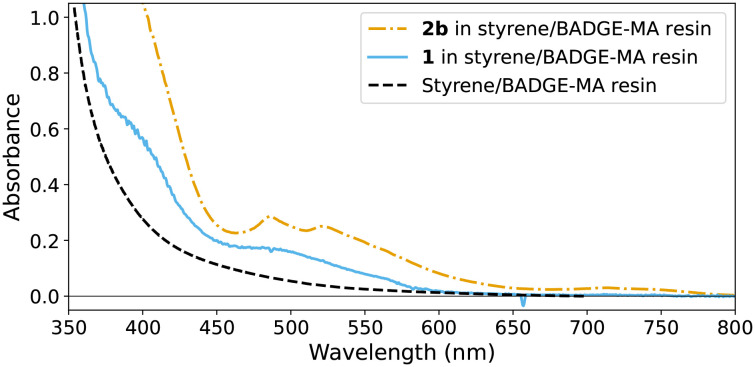
UV/vis absorption spectra of 0.5 mM 2b and of 0.5 mM 1 in BADGE-MA/styrene, and the absorption spectrum of BADGE-MA/styrene.

The UV/vis absorption spectra of the resin mixture and the resin mixture with 1 or 2b show the expected characteristic bands (Fig. 7),^15,26,27,37^ and are sufficiently distinct for them to be identified in the present reaction mixtures at the concentrations used (*ca.* 1 mM). Of particular note with respect to the discussion below is the characteristic sharp features of 2b at 460 and 520 nm, as well as the generally higher absorptivity at 400 nm compared with 1. In contrast, Mn(ii) complexes of tmtacn, such as 3a (Fig. 1) are colourless.^23,37^ Hence, it is expected that changes to the redox state of 1 and 2b can be monitored in real-time by UV/vis spectroscopy. It should be noted that although EPR spectroscopy at 77 K is generally useful in the study of manganese complexes, the complexes 1, 2b and 2a are EPR silent, and the EPR signals expected for Mn(ii) complexes of tmtacn,^16^ are overwhelmed by the organic radicals present in cured resins.^14^

### Catalyst ageing

**Fig. 8:**
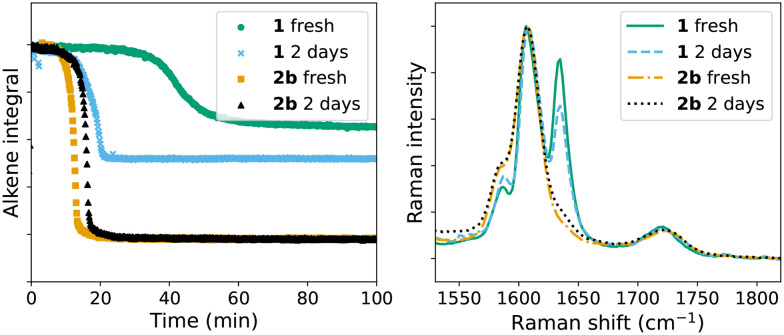
Left: Conversion of alkene during polymerisation (area (alkene integral) of the alkene C=C stretch band at 1630–1637 cm^−1^) with the initiator cumene hydroperoxide added directly after mixing of catalyst (1 (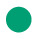), 2b (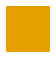)) with the resin, and initiated with cumene hydroperoxide added 2 days after mixing of catalyst (1 (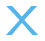), and 2b (▲)) with the resin. Right: Raman spectra 100 min after addition of cumene hydroperoxide for each reaction show differences in extent of polymerisation (loss of intensity at 1630–1637 cm^−1^) of alkene.

**Fig. 9:**
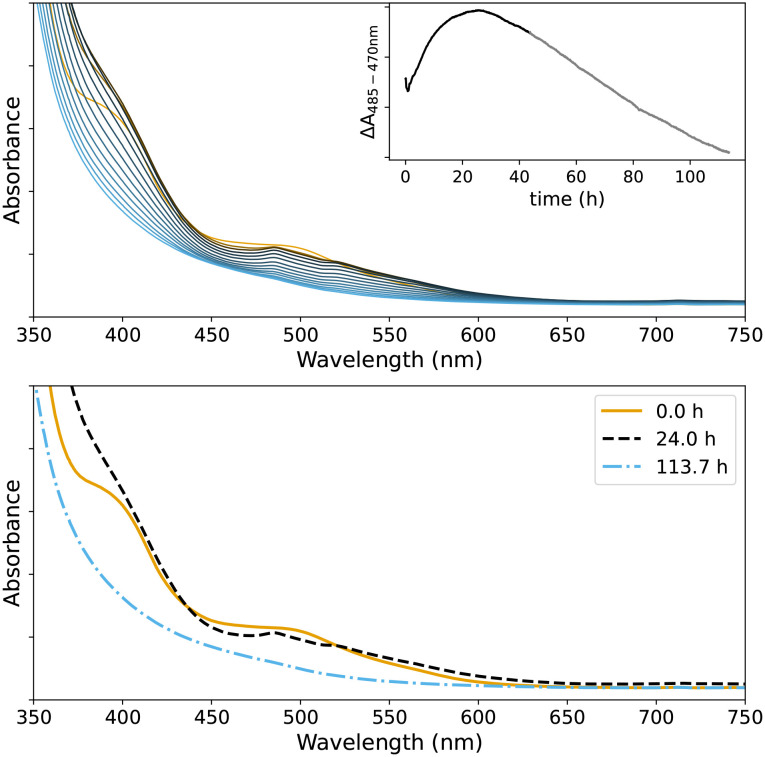
Top: UV/vis absorption spectrum of 1 in styrene/BADGE-MA over time at room temperature with difference in absorbance at 485 nm with respect to the absorbance at 470 nm shown as an insert, which indicates conversion of 1 to a Mn(iii) bis-carboxylato bridged species analogous to 2b. Bottom: UV/vis absorption spectra at selected time points showing the initial spectrum of 1 (orange), of a 2b type species (black) and eventual loss of visible absorbance (blue).

A lag period before the onset of polymerisation is observed with 1, which is comparable to that with Co(ii)(2-ethylhexanoate)_2_. However, when the resin containing 1 is stored for 2 days before addition of cumene hydroperoxide, the duration of lag period decreased to close to that obtained with 2b (from *ca.* 40 min, to <20 min). Indeed it became even shorter when the mixture 1/styrene/BADGE-MA was stored for several days more prior to addition of cumene hydroperoxide. Furthermore, the extent of conversion of alkene increased (Fig. 8), which is consistent with the exotherm produced by a more rapid reaction in the autoacceleration phase (*vide supra*). This change in behaviour on storage indicates that the catalyst converts to a new species, which reacts readily with cumene hydroperoxide, in the resin over time. UV/vis absorption spectroscopy shows that storage at room temperature of a mixture of 1/styrene/BADGE-MA shows initially a change in the spectrum of 1 to one that is similar to that of 2b, and thereafter a general decrease in visible absorption is observed (Fig. 9).

**Fig. 10:**
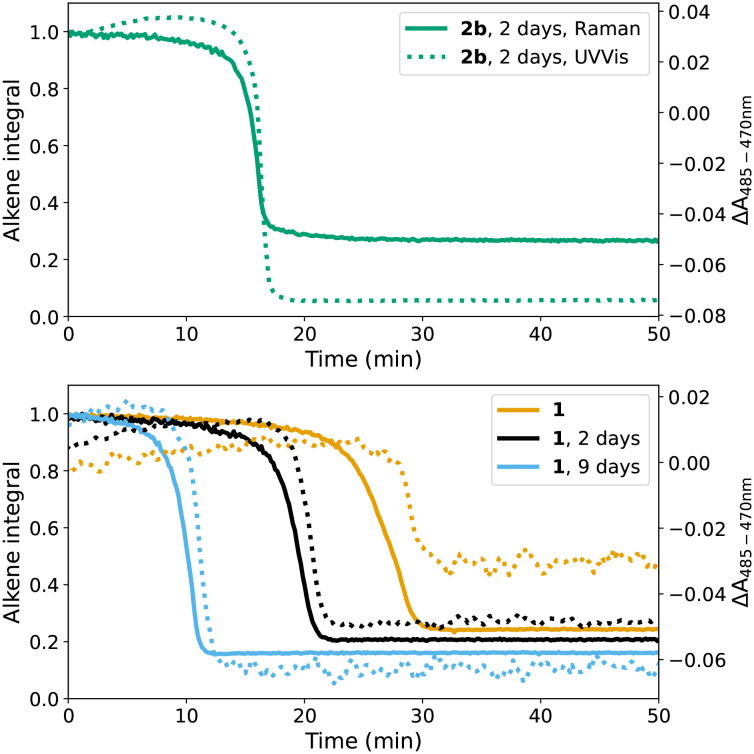
Raman (785 nm) intensity and change in absorbance during curing (top) initiated with 2b/cumene hydroperoxide; 2b/styrene/BADGE-MA was stored for 2 days at room temperature before addition of oxidant, (bottom) initiated with 1/cumene hydroperoxide, where initiation takes place immediately (orange), 2 days (black), and 9 days (blue) after addition of catalyst to the resin. Dashed spectra indicate change in visible absorbance and solid lines indicate intensity of C=C stretch Raman band. The Raman and UV/vis spectra are shown in Fig. S8.†

UV/vis absorption spectra of the resins, containing either 1 or 2b change suddenly, during curing with cumene hydroperoxide, concomitant with the onset of the autoacceleration phase (*i.e.* the period of rapid decrease in Raman intensity at *ca.* 1650 cm^−1^) regardless of the number of days between preparation of the resin and addition of cumene hydroperoxide (Fig. 10). Apart from a change in the baseline (not shown) due to a sudden increase in scattering of light by the sample, the change in absorbance of the characteristic bands of 1 at 485 and 520 nm coincides with changes observed by Raman spectroscopy. Two minor bands at 460 and 470 nm appear simultaneously, indicating the opening of the μ-oxo-bridge to form a species similar to 2b.^15^

**Fig. 11:**
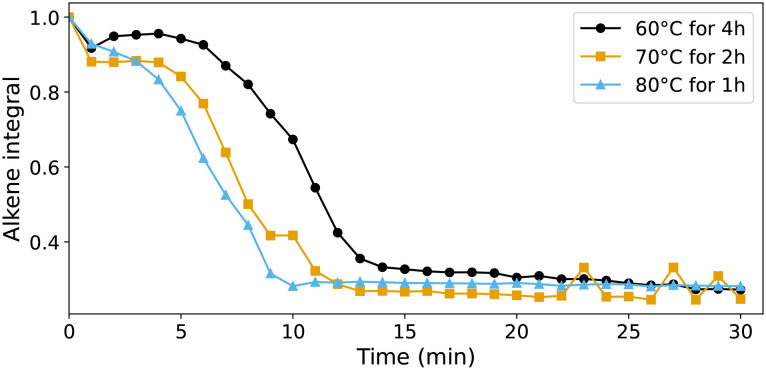
Integrated intensity of C=C stretching Raman (785 nm) band at 1630 cm^−1^ over time during the curing of BADGE-MA/styrene resin with 1 (0.5 mM), which had been held at 60–80 °C for several hours prior to addition of oxidant at room temperature.

**Fig. 12:**
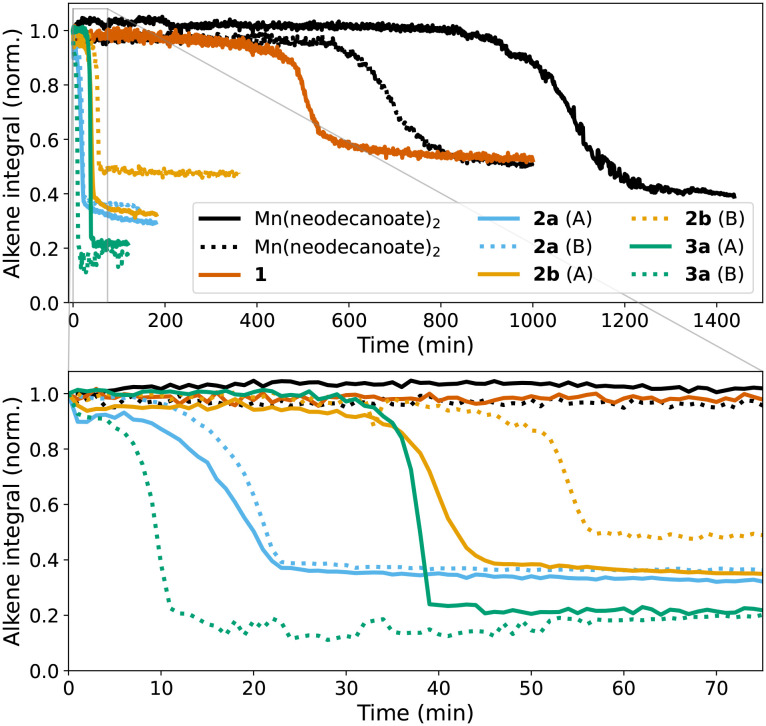
Integrated intensity of C=C stretching Raman band at 1630 cm^−1^ (*λ*_exc_ 785 nm) over time during the curing of BADGE-MA/styrene resin with Mn(ii) neodecanoate, 1, 2a (*in duplo*, A and B), 2b (A and B), and 3a (A and B).

The ageing of styrene/BADGE-MA resin containing 1 was accelerated thermally by preheating at either 60, 70, or 80 °C. At 60 °C, *in situ* monitoring by UV/vis absorption spectroscopy showed decolouration, *i.e.* reduction of 1, over several hours (Fig. S9†). Once cooled to room temperature, addition of cumene hydroperoxide was followed, in all cases, by a lag time before the onset of polymerisation that was shorter than observed even with 2b (Fig. 11). These data indicate that 1 and 2b are precursors to the complexes that activate cumene hydroperoxide in the resins, which are less coloured and hence most likely in the Mn(ii) oxidation state. Indeed comparison of the Mn(iii) and Mn (ii) complexes, 2a and 3a, respectively show that the shortest lag period is obtained with 3a, which is itself as short as an *in situ* prepared catalyst, *vide infra*. Furthermore the lag periods are less easily reproduced from batch to batch reflecting the sensitivity of the reaction to the concentration of manganese complex, Fig. 12.

### *In situ* catalyst preparation from Mn(ii) and ligand

**Fig. 13:**
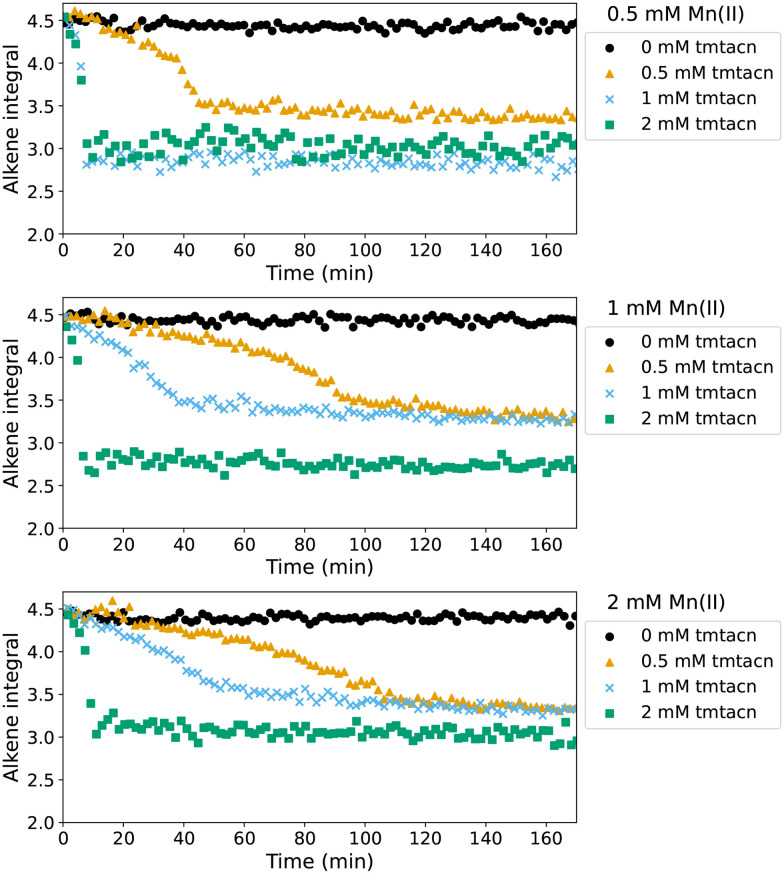
Rate of polymerisation initiated with Mn(neodecanoate)_2_/tmtacn/cumene hydroperoxide for Mn(neodecanoate)_2_ concentrations 0.5 mM (top), 1.0 mM (middle), and 2.0 mM (bottom) and tmtacn concentrations 0 mM (●), 0.5 mM (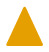), 1.0 mM (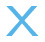), and 2.0 mM (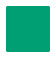).

Simpson *et al.* reported that the curing of alkyd coatings (with molecular oxygen as oxidant) proceeded more rapidly with a catalyst prepared *in situ* by mixing Mn(neodecanoate)_2_ and the ligand tmtacn than when using complex 1.^43^ These observations prompted us to compare the reactivity of preformed complexes with mixtures of Mn(ii) and the ligand tmtacn in curing alkene based resins with cumene hydroperoxide. As discussed above and earlier,^14^ Mn(neodecanoate)_2_ is able to initiate curing of these resins but with an undesirably long lag period (Fig. 12). Nevertheless, autoacceleration was observed even after lag periods of up to several hours, with only a modest dependence on concentration of Mn(neodecanoate)_2_. Curing of resins with 0.5–2 mM of Mn(neodecanoate)_2_ and a 0.5–2 mM tmtacn (Fig. 13) show in all cases a lag period followed by autoacceleration. The lag period was much shorter than with Mn(neodecanoate)_2_ alone. Notably even with an excess of manganese with respect to the tmtacn ligand added, the lag period is decreased substantially compared to without ligand present, Fig. 13. With an excess of tmtacn, with respect to Mn(neodecanoate)_2_, curing is complete within a few minutes, and the rate and conversion is greater than that observed with 1 or 2b. Curing profiles close to that observed with Co(ii)(2-ethylhexanoate)_2_ are achieved with 0.5 mM of tmtacn ligand, and 1 or 2 mM of Mn(neodecanoate)_2_ showing that *in situ* catalyst preparation can provide for equivalent curing behaviour to the reference Co(ii)(2-ethylhexanoate)_2_/cumene hydroperoxide initiated polymerisation. A challenge, however, is that minor fluctuations in manganese or ligand concentration, as well as in other parameters such as resin composition and temperature, significantly alter the curing profile and final extent of conversion. Hence, the same curing profile and extent of curing can be achieved but without the robustness to small variations in catalyst composition found in the cobalt system.

It is of particular note that the reaction proceeded rapidly with a short lag time where the concentration of the ligand was greater than that of the Mn(neodecanoate)_2_, regardless of the absolute concentration. This effect cannot be ascribed to competition for cumene hydroperoxide, since Mn(neodecanoate)_2_ alone does not decompose the latter more rapidly (*vide supra*). It may point, however, to the need to form multinuclear complexes with the ligand tmtacn to react with the cumene hydroperoxide productively (*i.e.* generate radicals).

## Discussion

Complex 1 has been applied widely with H_2_O_2_ in the epoxidation and dihydroxylation of alkenes, alcohol, aldehyde, and C–H oxidations.^5,16^ The reduction of 1 and subsequent ligand exchange reactions to form, *e.g.*, 2b and 2a, are key to its catalytic activity in these reactions. The reduction is autocatalytic and the lag periods observed in oxidations with H_2_O_2_ are due to waiting for reduction of 1 (Fig. 1).^37^

Indeed, in the oxidation of alkenes with H_2_O_2_, a lag period is not observed when 2a, 2b or 3a are used as catalyst. The reduction/activation of 1 can therefore be circumvented by mixing the ligand tmtacn with a Mn(ii) salt *in situ*, or by use of the Mn(ii) and Mn(iii) complexes such as 3a and 2a, Fig. 1.^15,21,23,40^

A peculiar aspect of the chemistry of 1 is that H_2_O_2_ acts as a two electron reductant under acidic catalytic conditions.^15,23^ This catalyst activation step is not achieved with alkyl hydroperoxides, however, as they are not able to reduce 1. Indeed in the oxidation of benzyl alcohol with *t*BuOOH reported by Zondervan *et al.*,^16,24,30^ activity was only observed when preactivation of 1 was carried out with H_2_O_2_. Later, de Boer showed that *t*BuOOH could be used without catalyst preactivation, with the already reduced (relative to 1) complex 2a, albeit with much lower extent of oxidation of alkene than with H_2_O_2_ as oxidant.^24^

The composition of the resin used should be considered in light of earlier studies where carboxylic acids were required to form, *e.g.*, 2b, from 1.^15,23^ The BADGE-MA crosslinking monomer, used here, was prepared earlier with known composition^14^ and has an acid value that equates to *ca.* 0.1 molal acid content, mostly methacrylic acid from the synthesis, which provides carboxylato ligands for the manganese complexes studied here.^23^ In addition, the resin contains stabilisers including butylated hydroxytoluene (BHT) and a small amount of acetonitrile or other non-reactive diluent used to dose the reaction mixture with catalyst. All of these components can potentially interact chemically with the manganese catalysts and influence the reactions driven by changes in redox state. Indeed, in the present study it is clear that reducing agents (*e.g.*, BHT) together with acids present in the resins facilitate the reduction of 1 to an intermediate Mn(iii) carboxylato bridged complex (*i.e.* similar to 2a/2b) and ultimately to a colourless complex, likely in the Mn(ii) oxidation state, over time prior to addition of cumene hydroperoxide.

**Scheme 1:**
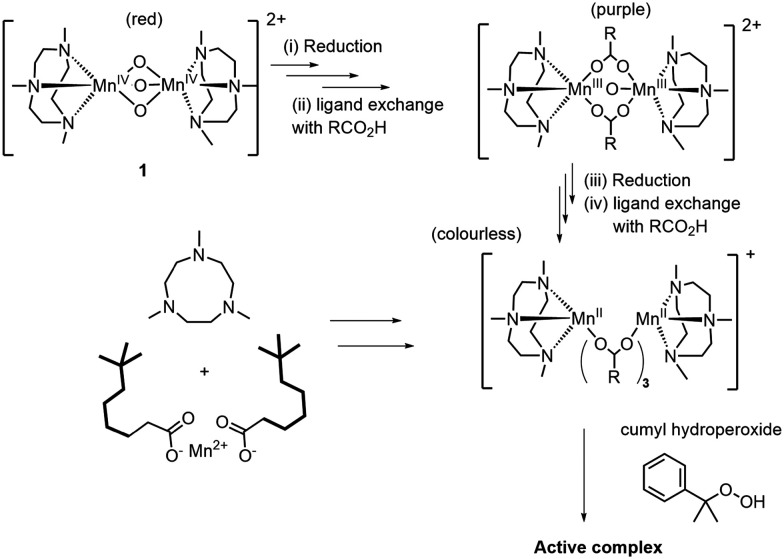
Reduction of 1 to 2 (where RCO_2_H is residual methacrylic acid), is followed by further reduction to the manganese(ii) oxidation state and exchange of the μ-oxo bridges of 2 carboxylate ligands. The same species can possibly be formed in situ by mixing Mn(ii)(neodecanoate)_2_ with tmtacn. Addition of cumene hydroperoxide forms the active species in the reaction.

Overall, it can be concluded that the differences in lag-period observed for the various manganese complexes is due to the time taken for them to undergo reduction to lower oxidations states with the ligand exchange reactions accompanying the change. Indeed, with 3a or mixtures of Mn(ii)(neodecanoate)_2_ and the ligand tmtacn, the lag time was reduced to several minutes. In contrast to oxidation reactions catalysed by 1 with H_2_O_2_ (*vide supra*), a lag period remains in alkene polymerisation due to the time taken to reach the gel point in the polymerisation. The various processes are described in Scheme 1.

## Conclusion

Cobalt-free alternatives for BADGE-MA/styrene curing requires three attributes: i) the same final chemical and physical properties of coating; ii) the same curing time profile with window for application before gelation and autoacceleration; and iii) the same robustness to variations in composition and catalyst concentrations especially. The use of manganese based catalysts for the replacement of cobalt alkyl carboxylates for initiation of radical polymerisation of alkene based resins was explored using styrene/BADGE-MA as representative of alkene based resins. It is clear that while Mn(neodecanoate)_2_ is able to activate cumene hydroperoxide to initiate polymerisation, it is much too slow and thus not a viable alternative. The polydentate amine ligand tmtacn, added either separately to the reaction mixture with Mn(neodecanoate)_2_ or as preformed complexes, [Mn^III,III^_2_(μ‐O)(CH_3_CO_2_)_2_(tmtacn)_2_](PF_6_)_2_ (2b), [Mn^IV,IV^_2_(μ‐O)_3_(tmtacn)_2_](PF_6_)_2_ (1) *etc.*, both shortens the lag period and significantly enhances the rate and extent of polymerisation (due to reaction exothermicity), with in some cases almost full conversion. The increased conversion compared with that obtained with cobalt soap is, however, undesirable as it significantly alters the resins mechanical properties. For coating applications, this aspect is less important but when applied as thick (>0.5 cm) coatings heat dissipation should be considered.

It cannot be concluded with certainty that the same catalyst is formed (*i.e.* oxidation state and coordination environment) when a preformed complex or a mixture of Mn(neodecanoate)_2_ and tmtacn is used to catalyse cumene hydroperoxide decomposition. However, it is likely to be the case, as the reduction in the lag period observed when the resin containing 1 was stored for some time, or heat treated, before addition of oxidant indicates. Notably the minor changes in UV/vis absorption spectra of resins containing 1, 2a, *etc.* before the onset of autoacceleration, indicates that a minor amount of complex in the active form is responsible for initiation, while most of the complex remains inactive.

In conclusion, manganese/tmtacn based catalysts can achieve a sigmoidal curing profile and extent of conversions required as replacement for Co(ii)(2-ethylhexanoate)_2_. Indeed, 1 shows a comparable lag period before the onset of autoacceleration. Future studies will focus on understanding the reaction(s) that trigger catalytic activity and the mode of action of both cobalt and manganese in activating cumene hydroperoxide.

## Author contributions

All authors contributed equally to the design of the study. L. E. E., A. S. S., E. G. S., Y. Z., H. d. B. performed the experiments and analysed the data. L. E. E. and W. R. B. wrote the manuscript. All authors read and commented on the results and manuscript.

## Conflicts of interest

There are no conflicts to declare.

## Supplementary Material

CY-014-D4CY00042K-s001
