# Supplementary material for: Activation of alkyl hydroperoxides by manganese complexes of tmtacn for initiation of radical polymerisation of alkenes
Source: Catal Sci Technol. 2024 Mar 13;14(9):2423–33. doi: 10.1039/d4cy00042k (PMC11075131; doi:10.1039/d4cy00042k)
Supplement: CY-014-D4CY00042K-s001 [file CY-014-D4CY00042K-s001.pdf]

Electronic Supplementary Material (ESI) for Catalysis Science & Technology.
This journal is © The Royal Society of Chemistry 2024

Electronic Supplementary Information

Activation of alkyl hydroperoxides by manganese complexes of tmtacn for initiation of radical polymerisation of alkene

Linda E. Eijsink,[a] Andy S. Sardjan,[a] Esther G. Sinnema,[a] Hugo den Besten,[a] Yanrong Zhang,[a] Ronald Hage,[a] Keimpe J. van den Berg,[b] Jitte Flapper,[c] Ben L. Feringa,[a] and Wesley R. Browne*[a]

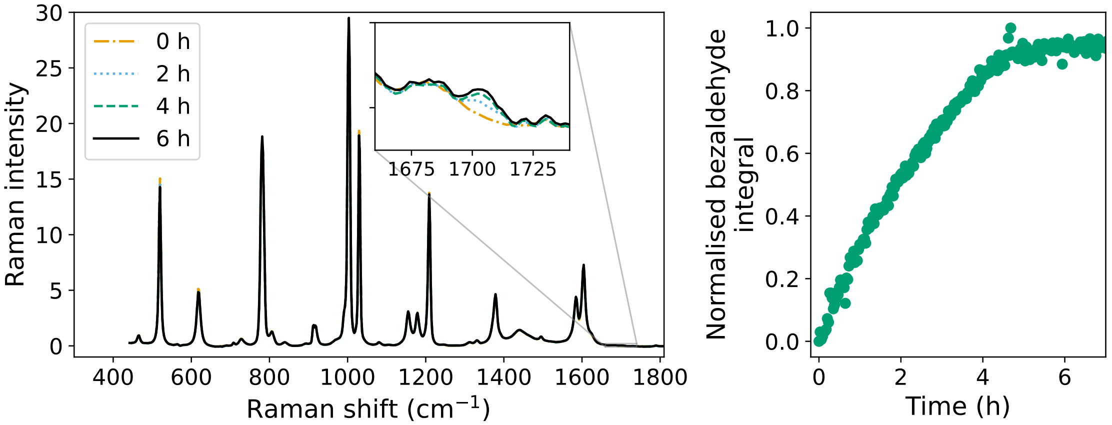

Figure S1 Raman spectra of a reaction mixture containing cumene hydroperoxide in toluene with 2b (added as a solution in acetonitrile (50% final concentration)) at selected times. The appearance of a characteristic band of benzaldehyde at 1702 cm^{-1} is shown in the inset and the intensity of the Raman band over time shown on the right.

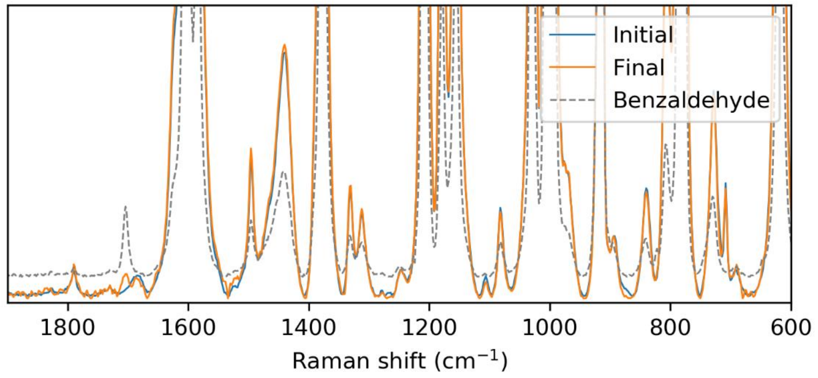

Figure S2 The initial and final spectra from Figure S1 and Raman spectrum of a solution of benzaldehyde (50 mM) in toluene, with the same amount of acetonitrile present in reaction mixture).

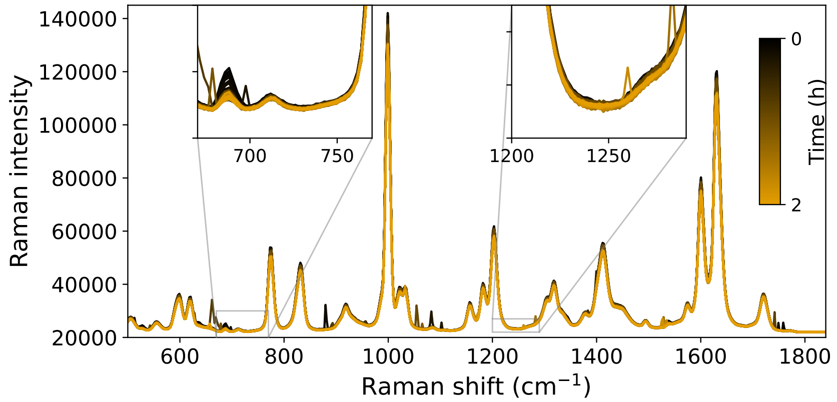

Figure S3 Styrene/MMA mixture over time following addition of cumene hydroperoxide with 2b. The sharp band at 687 cm^{-1} originates from room lights, as do the bands at 1266, 1344, 1538 cm^{-1}.

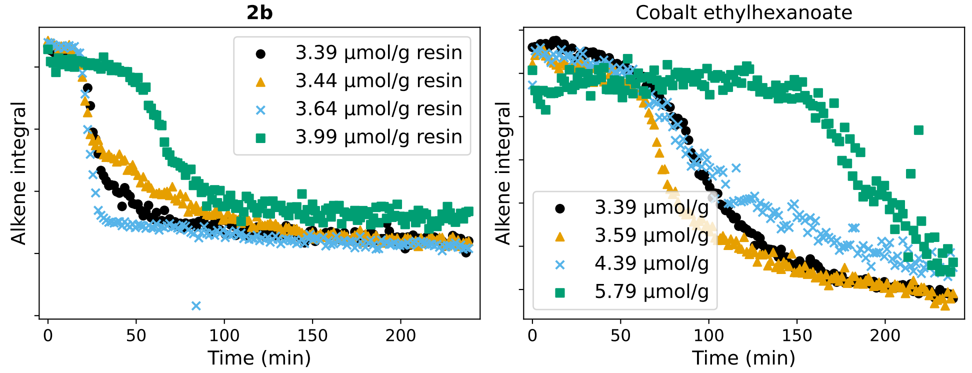

Figure S4 Addition of inhibitor BHT to (left) 2b, and (right) Co(II)(2−ethyl−hexanoate)$_2$ catalysed BADGE-MA/styrene polymerisation. The initial concentration of BHT present in the resin is 3.39 μmol/g.

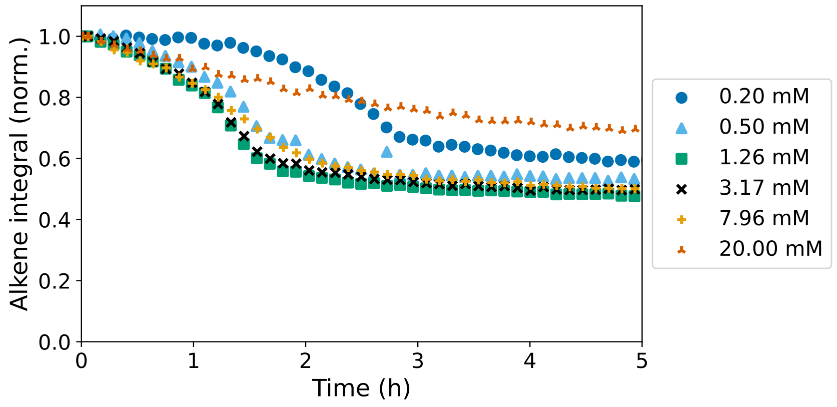

Figure S5 Conversion of alkene during polymerisation of styrene/BADGE-MA initiated by the decomposition of cumene hydroperoxide by between 0.2 and 20 mM Co(II)(2−ethylhexanoate)$_2$ monitored by Raman spectroscopy (λ_{exc} 785 nm).

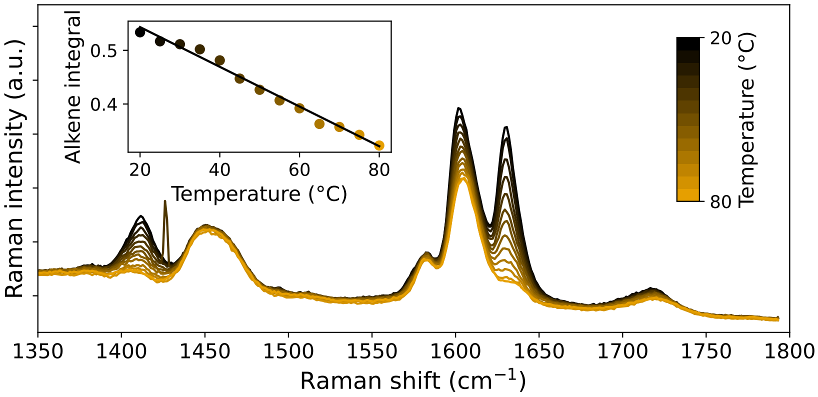

Figure S6 Styrene/BADGE-MA was cured with cumene hydroperoxide and Co(II)(2−ethylhexanoate)$_2$ (1.88 mM) at 20 °C for 3 h followed by post-curing to 80 °C (5 °C steps with 10 min intervals between steps). Initial Raman spectrum (after curing at 20 °C to spectrum at 80 °C is shown as black to orange. The area (Alkene integral) of the alkene C=C stretch band at 1630-1637 cm^{-1} at each temperature is shown as an inset.

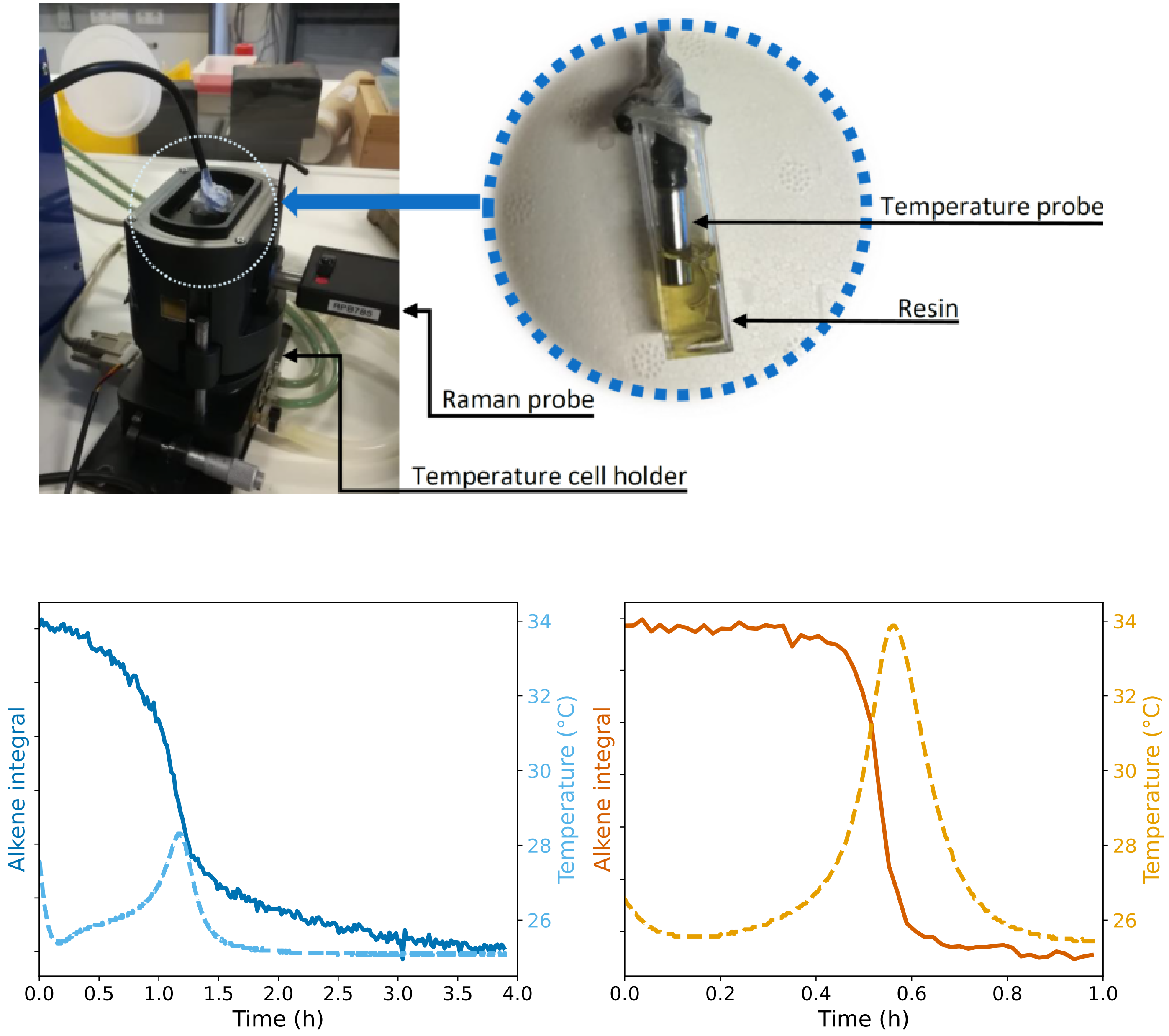

Figure S7 Top: Instrumental arrangement for simultaneous recording of Raman spectra and temperature during curing. The temperature in the resin is monitored using a termistor. Bottom left: Integrated area of Raman band of styrene (C=C stretch, dark blue) and internal temperature (light blue, dashed) for polymerisation initiated by Co(II)(2−ethylhexanoate)$_2$ catalysed decomposition of cumene hydroperoxide. Bottom right: Conversion (red) and internal temperature (orange, dashed) for polymerisation initiated by 2b catalysed decomposition of cumene hydroperoxide.

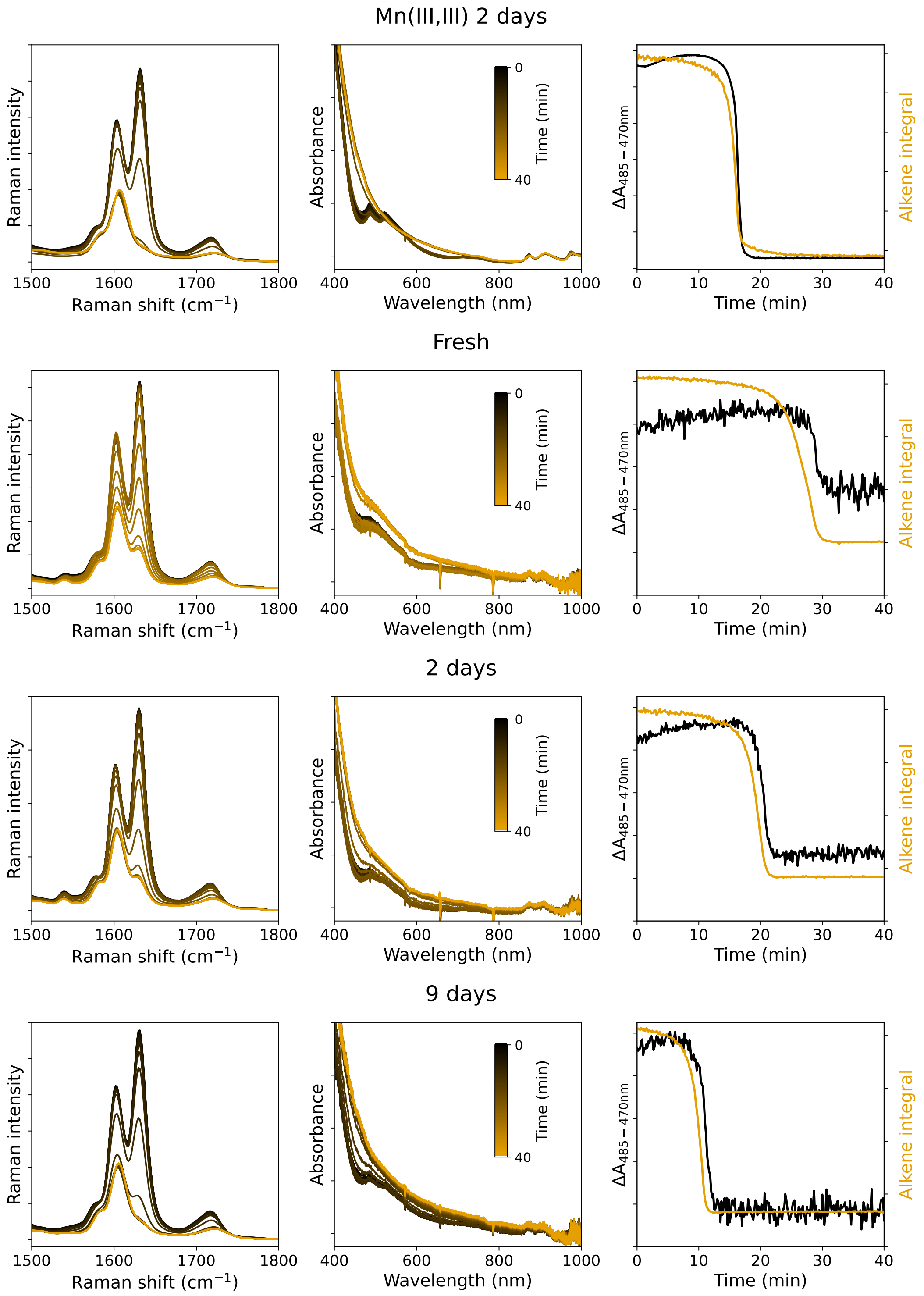

Figure S8 Raman and UV/vis absorption spectroscopy during curing initiated by addition of cumene hydroperoxide, with 2b, initiation after 2 days (top), with 1 and immediate initiation (2nd down), with 1, initiation after 2 days (3rd down), and with 1, initiation after 9 days (bottom). Raman intensity at 1630 cm^{-1} (red) and average absorbance between 475 to 485 nm (black) is indicated.

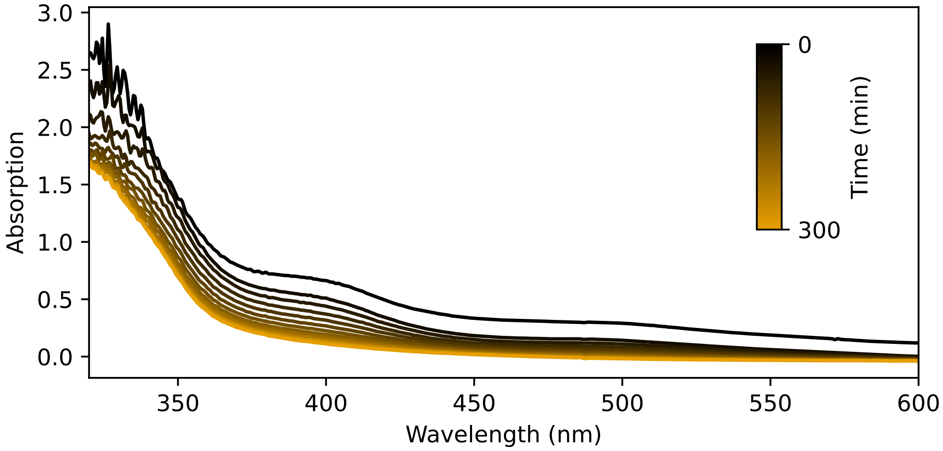

Figure S9 UV/vis absorption spectroscopy over time of BADGE-MA/styrene resin with 1 (0.5 mM) at 60° C showing decrease in absorbance at all wavelengths.
